# Dataset on the generation of red-kinked current-voltage curves in Cu(In,Ga)Se_2_ solar cells due to buffer/window interfacial defects

**DOI:** 10.1016/j.dib.2019.104503

**Published:** 2019-09-12

**Authors:** Choong-Heui Chung

**Affiliations:** Department of Materials Science and Engineering, Hanbat National University, Daejeon, 34158, Republic of Korea

**Keywords:** Solar cells, CIGS, Red kink, Buffer/window interface, SCAPS-1D

## Abstract

Red-kinked current-voltage characteristics in silver nanowire transparent electrode based Cu(In,Ga)Se_2_ solar cells have been reported [1–3]. The author has recently revealed that the buffer/window interfacial defects cause the generation of red-kinked current-voltage characteristics in the solar cells [1]. This article provides the dataset regarding the red-kink for Cu(In,Ga)Se_2_ solar cells as a function of the donor density in n-type window and CdS buffer/window interfacial defect density. The data were obtained by the simulation for Cu(In,Ga)Se_2_ solar cells using SCAPS-1D. The data include current density-voltage curves, fill factor, open-circuit voltage, short-circuit current density, and efficiency in the solar cells, and energy band bending in the Cu(In,Ga)Se_2_ layer.

**Table:** Specifications Table

Subject	Electrical engineering
Specific subject area	Solar cells
Type of data	Tables and Figures
How data were acquired	SCAPS-1D simulation
Data format	Raw, and analyzed
Parameters for data collection	The donor density in the window layer, and CdS buffer/window interfacial defect density
Description of data collection	The data including current-voltage curves, fill factor, open-circuit voltage, short-circuit current density, and efficiency in the Cu(In,Ga)Se_2_ solar cells, and energy band bending in the Cu(In,Ga)Se_2_ layer were obtained by SCAPS-1D simulation for Cu(In,Ga)Se_2_ solar cells
Data source location	Hanbat National University, Daejeon 34158, Republic of Korea
Data accessibility	Raw data related to Fig. 1, Fig. 2, Fig. 3 are available within this article as a supplementary file.
Related research article	Author's name: Jiseong Jang et al. Title: Cu(In,Ga)Se_2_ thin film solar cells with solution processed silver nanowire composite window layers: Buffer/window junctions and their effects Journal: Solar Energy Materials and Solar Cells https://doi.org/10.1016/j.solmat.2017.05.051

**Table:** 

**Value of the Data** • Silver nanowire transparent electrodes have a potential to replace high cost transparent conducting oxides, and they have been employed to solar cells. However, incomplete contact between silver nanowires and the devices could cause red-kinked current-voltage characteristics in the solar cells. The data provide the effect of materials properties on the red-kink. Therefore, the data could guide researchers to tailor materials properties in right direction. • The data can be useful for device/process engineers who are responsible for determining materials and structure of a window layer for a solar cell. • Tandem solar cell, multi-junction solar cells consisting of two different materials having considerably different band gaps, have recently been studied. When a Cu(In,Ga)Se_2_ (CIGS) solar cell is employed as a bottom cell, only long wavelength-light (i.e. red-light) illuminates the CIGS solar cell, which could cause a red-kink in the tandem solar cells. The data would help to develop an interlayer between a bottom and a top cell of the tandem solar cells.

## 1 Data

Kink in a current density – voltage (*J-V*) curve is a substantial decrease of photocurrent in a certain forward bias region in a solar cell. The CIGS solar cells, based on the standard structure of CIGS/CdS p-n junction, often show the kink under red-light, whose phenomenon is called as red-kink. The red kink can be caused by the CdS buffer/window interfacial defects [1], [2].

**Fig. 1:**
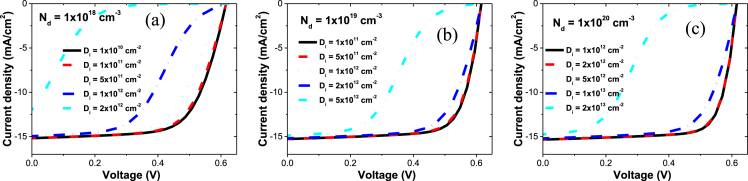
Current density – voltage (*J-V*) curves for CIGS solar cells as a function of D_i_ at the N_d_ values of (a) 1ⅹ10^18^, (b) 1ⅹ10^19^, and (c) 1ⅹ10^20^ cm^−3^.

Fig. 1 shows *J-V* curves for CIGS solar cells as a function of the shallow donor density (N_d_) in the n-type window and buffer/window interfacial defect density (D_i_). The degree of the red kink is known to be affected by the both N_d_ and D_i_ values [1]. At a fixed N_d_ value, the degree of the red kink is increased with increasing the D_i_ value. To be free from the red kink, the D_i_ values have to be kept below 1 × 10^11^, 1 × 10^12^, and 5 × 10^12^ cm^−2^ at the N_d_ value of 1 × 10^18^, 1 × 10^19^, and 1 × 10^20^ cm^−3^, respectively. The experimental control and measurement of D_i_ in CIGS solar cells would be difficult. The comparison between the measured *J-V* and the simulated *J-V* would be useful to estimate D_i_ based on the measured N_d_. An experimental validation to the simulated values can be found in its related article [1].

**Fig. 2:**
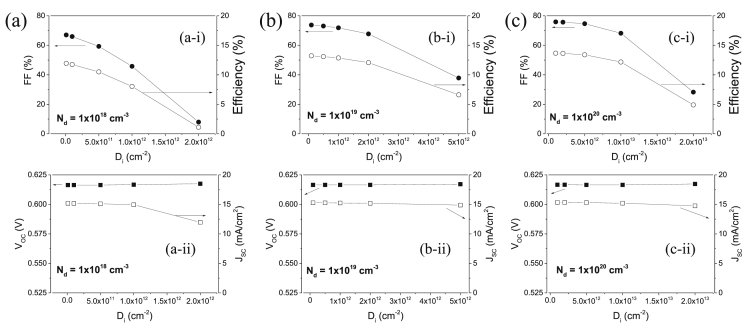
Solar cell parameters for the CIGS solar cells as a function of D_i_ at the N_d_ values of (a) 1ⅹ10^18^, (b) 1ⅹ10^19^, and (c) 1ⅹ10^20^ cm^−3^. (i) FF (%) and Efficiency (%). (ii) V_OC_ (V) and J_SC_ (mA/cm^2^).

Fig. 2 shows solar cell parameters including fill factor (FF), open-circuit voltage (V_OC_), short-circuit current density (J_SC_), and efficiency obtained from the simulated J-V curves of Fig. 1. Fig. 2a shows FF and efficiency (a-i), and V_OC_ and J_SC_ (a-ii) as a function of D_i_ at the N_d_ value of 1 × 10^18^ cm^−3^. Fig. 2b shows FF and efficiency (b-i), and V_OC_ and J_SC_ (b-ii) as a function of D_i_ at the N_d_ value of 1 × 10^19^ cm^−3^. Fig. 2c shows FF and efficiency (c-i), and V_OC_ and J_SC_ (c-ii) as a function of D_i_ at the N_d_ value of 1 × 10^20^ cm^−3^. The V_OC_ and J_SC_ values are almost not affected by the values of N_d_ and D_i_, but the FF values are significantly decreased with increasing the D_i_ value for all the investigated N_d_ values. The efficiencies thus are limited by the decreased FF values.

**Fig. 3:**
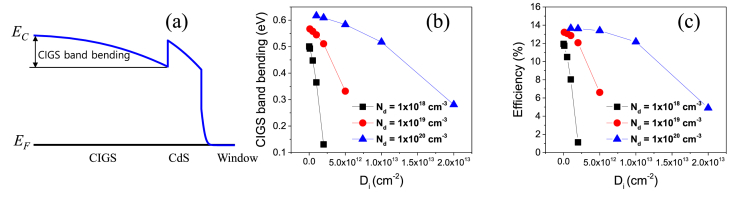
(a) Energy band alignment of a CIGS solar cell at thermal equilibrium showing CIGS band bending. (b) The CIGS band bending as a function of D_i_ at the N_d_ values of 1ⅹ10^18^, 1ⅹ10^19^, and 1ⅹ10^20^ cm^−3^. (c) The efficiencies of CIGS solar cells as a function of D_i_ at N_d_ values of 1ⅹ10^18^, 1ⅹ10^19^, and 1ⅹ10^20^ cm^−3^.

Fig. 3a illustrates the energy band alignment of a CIGS solar cell at thermal equilibrium showing CIGS band bending. Fig. 3b shows the CIGS band bending values as a function of D_i_ at the N_d_ values of 1ⅹ10^18^, 1ⅹ10^19^, and 1ⅹ10^20^ cm^−3^. Fig. 3c shows the efficiencies of CIGS solar cells as a function of D_i_ at the N_d_ values of 1ⅹ10^18^, 1ⅹ10^19^, and 1ⅹ10^20^ cm^−3^. The variation of the efficiency is very similar to that the CIGS band bending with the values of N_d_ and D_i_.

Raw data related to Fig. 1, Fig. 2, Fig. 3 are available within this article as a supplementary file.

## 2 Experimental design, materials, and methods

Simulated CIGS solar cells have a structure of window/CdS buffer/CIGS/rear contact for SCAPS-1D simulation. SCAPS-1D (Solar Cell Capacitance Simulator) is a one dimensional solar cell simulation program developed at the Department of Electronics and Information Systems of the University of Gent, Belgium (http://scaps.elis.ugent.be/). Table 1 summarizes input parameters of p-type CIGS, n-type CdS buffer, and n-type window layer, respectively, for SCAPS-1D simulation of the CIGS solar cells. Table 2 summarizes operation conditions for the simulation of the CIGS solar cells. The input parameters shown in Table 1, Table 2 are in the ranges where previous reports simulated CIGS solar cells [3], [4], [5].

**Table 1:** Input parameters for SCAPS-1D simulation for CIGS solar cells with CdS buffer/window interfacial defects.

Parameters	p-type CIGS	n-type CdS	n-type Window
thickness (μm)	2	0.05	0.5
band gap (eV)	1.15	2.4	3.7
electron affinity (eV)	4.6	4.4	4.7
relative dielectric constant	13.6	10	9.0
conduction band effective density of states (cm^−3^)	2.2 × 10^18^	2.2 × 10^18^	2.2 × 10^18^
valence band effective density of states (cm^−3^)	1.8 × 10^19^	1.8 × 10^19^	1.8 × 10^19^
shallow donor or acceptor density (cm^−3^)	1.0 × 10^16^	1.0 × 10^15^	Variation

**Table 2:** Operation conditions for the simulation of CIGS solar cells.

Operating condition	Value
Light source	AM 1.5G
Spectrum cut off	Wavelength below 700 nm
Cut off spectrum power (mW/cm^2^)	525.3
Temperature (K)	300

The N_d_ value can be varied from 1 × 10^18^ to 1 × 10^21^ cm^−3^ depending on the preparation conditions of the n-type windows [3], [6]. The D_i_ values are typically larger than ∼10^10^ cm^−3^, and smaller than ∼10^13^ cm^−3^ [7]. At the N_d_ value of 1 × 10^21^ cm^−3^, a kinked *J-V* was not observed for the typical D_i_ ranges in the simulation. Thus, the simulated *J-V* curves are shown for N_d_ = 1 × 10^18^ (Fig. 1a) 1 × ×10^19^ (Fig. 1b), and 1 × 10^20^ cm^−3^ (Fig. 1c) for the above D_i_ ranges. The light source is AM1.5 G, and light wavelength below 700 nm is cut off (Table 2).
