# Supplementary material for: Dataset on the generation of red-kinked current-voltage curves in Cu(In,Ga)Se2 solar cells due to buffer/window interfacial defects
Source: Data Brief. 2019 Sep 12;26:104503. doi: 10.1016/j.dib.2019.104503 (PMC6811924; doi:10.1016/j.dib.2019.104503)
Supplement: Multimedia component 1 [file mmc1.pdf]

Fig. 1(a)

$N_d=1\times10^{18}$ cm^{-3}									
$D_i=1\times10^{10}$ cm^{-2}		$D_i=1\times10^{11}$ cm^{-2}		$D_i=5\times10^{11}$ cm^{-2}		$D_i=1\times10^{12}$ cm^{-2}		$D_i=2\times10^{12}$ cm^{-2}	
Voltage (V)	J (mA/cm^2)	Voltage (V)	J (mA/cm^2)	Voltage (V)	J (mA/cm^2)	Voltage (V)	J (mA/cm^2)	Voltage (V)	J (mA/cm^2)
-0.015	-15.197	-0.015	-15.185	-0.015	-15.115	-0.015	-14.970	-0.012	-12.457
0.005	-15.173	0.005	-15.161	0.005	-15.090	0.005	-14.942	0.008	-11.591
0.025	-15.150	0.025	-15.137	0.025	-15.065	0.025	-14.912	0.030	-10.494
0.045	-15.125	0.045	-15.113	0.045	-15.039	0.045	-14.882	0.051	-9.188
0.065	-15.101	0.065	-15.088	0.065	-15.012	0.065	-14.851	0.072	-7.747
0.085	-15.075	0.085	-15.062	0.085	-14.985	0.085	-14.818	0.094	-6.282
0.105	-15.049	0.105	-15.036	0.105	-14.957	0.105	-14.782	0.115	-4.910
0.125	-15.022	0.125	-15.009	0.125	-14.928	0.125	-14.743	0.136	-3.720
0.145	-14.995	0.145	-14.981	0.145	-14.897	0.145	-14.700	0.157	-2.749
0.165	-14.966	0.165	-14.952	0.165	-14.866	0.165	-14.651	0.178	-1.997
0.185	-14.937	0.185	-14.922	0.185	-14.832	0.185	-14.592	0.199	-1.435
0.205	-14.906	0.205	-14.891	0.205	-14.797	0.205	-14.518	0.219	-1.024
0.225	-14.875	0.225	-14.859	0.225	-14.759	0.226	-14.421	0.239	-0.728
0.245	-14.841	0.245	-14.825	0.245	-14.717	0.246	-14.288	0.259	-0.517
0.265	-14.806	0.265	-14.788	0.265	-14.668	0.266	-14.099	0.280	-0.367
0.285	-14.768	0.285	-14.748	0.285	-14.611	0.286	-13.828	0.300	-0.260
0.305	-14.725	0.305	-14.704	0.305	-14.540	0.307	-13.434	0.320	-0.184
0.325	-14.677	0.325	-14.653	0.326	-14.447	0.327	-12.867	0.340	-0.130
0.345	-14.621	0.345	-14.590	0.346	-14.320	0.348	-12.077	0.360	-0.091
0.365	-14.550	0.365	-14.511	0.366	-14.139	0.369	-11.022	0.380	-0.063
0.386	-14.456	0.386	-14.405	0.386	-13.874	0.390	-9.704	0.400	-0.044
0.406	-14.327	0.406	-14.255	0.407	-13.484	0.412	-8.187	0.420	-0.030
0.426	-14.140	0.426	-14.035	0.427	-12.914	0.433	-6.595	0.440	-0.021
0.446	-13.859	0.446	-13.705	0.448	-12.101	0.455	-5.077	0.460	-0.014
0.467	-13.432	0.467	-13.207	0.469	-10.993	0.476	-3.754	0.480	-0.009
0.487	-12.783	0.488	-12.462	0.490	-9.578	0.497	-2.684	0.500	-0.006
0.508	-11.812	0.509	-11.377	0.512	-7.913	0.518	-1.864	0.520	-0.004
0.530	-10.411	0.530	-9.868	0.534	-6.121	0.539	-1.257	0.540	-0.002
0.552	-8.486	0.552	-7.888	0.556	-4.348	0.559	-0.810	0.560	-0.001
0.574	-5.993	0.575	-5.453	0.577	-2.695	0.580	-0.474	0.580	-0.001
0.597	-2.936	0.597	-2.616	0.599	-1.182	0.600	-0.204	0.600	0.000
0.621	0.687	0.621	0.603	0.620	0.255	0.620	0.038	0.620	0.000
0.645	4.968	0.644	4.295	0.642	1.747	0.640	0.285	0.640	0.000
0.670	10.169	0.669	8.713	0.663	3.468	0.661	0.571	0.660	0.001
0.697	16.771	0.694	14.298	0.686	5.643	0.681	0.932	0.680	0.001
0.726	25.504	0.722	21.694	0.709	8.556	0.701	1.408	0.700	0.001
0.757	37.404	0.752	31.797	0.733	12.570	0.722	2.049	0.720	0.002
0.794	53.883	0.786	45.815	0.758	18.151	0.743	2.910	0.740	0.002
0.837	76.835	0.825	65.351	0.786	25.889	0.764	4.055	0.760	0.003

Fig. 1(b)

$N_d=1\times10^{19}$ cm^{-3}									
$D_i=1\times10^{11}$ cm^{-2}		$D_i=5\times10^{11}$ cm^{-2}		$D_i=1\times10^{12}$ cm^{-2}		$D_i=2\times10^{12}$ cm^{-2}		$D_i=5\times10^{12}$ cm^{-2}	
Voltage (V)	J (mA/cm^2)	Voltage (V)	J (mA/cm^2)	Voltage (V)	J (mA/cm^2)	Voltage (V)	J (mA/cm^2)	Voltage (V)	J (mA/cm^2)
-0.015	-15.289	-0.015	-15.277	-0.015	-15.259	-0.015	-15.212	-0.015	-14.903
0.005	-15.267	0.005	-15.255	0.005	-15.237	0.005	-15.188	0.005	-14.871
0.025	-15.245	0.025	-15.233	0.025	-15.214	0.025	-15.165	0.025	-14.838
0.045	-15.223	0.045	-15.210	0.045	-15.191	0.045	-15.140	0.045	-14.802
0.065	-15.200	0.065	-15.187	0.065	-15.167	0.065	-15.116	0.065	-14.764
0.085	-15.177	0.085	-15.163	0.085	-15.143	0.085	-15.090	0.085	-14.721
0.105	-15.153	0.105	-15.139	0.105	-15.119	0.105	-15.064	0.105	-14.673
0.125	-15.128	0.125	-15.114	0.125	-15.093	0.125	-15.038	0.125	-14.616
0.145	-15.103	0.145	-15.088	0.145	-15.067	0.145	-15.010	0.145	-14.544
0.165	-15.077	0.165	-15.062	0.165	-15.040	0.165	-14.982	0.166	-14.451
0.185	-15.050	0.185	-15.035	0.185	-15.013	0.185	-14.952	0.186	-14.323
0.205	-15.023	0.205	-15.007	0.205	-14.984	0.205	-14.922	0.206	-14.140
0.225	-14.995	0.225	-14.979	0.225	-14.955	0.225	-14.891	0.226	-13.871
0.245	-14.966	0.245	-14.949	0.245	-14.925	0.245	-14.858	0.247	-13.472
0.265	-14.936	0.265	-14.919	0.265	-14.893	0.265	-14.823	0.267	-12.888
0.285	-14.905	0.285	-14.887	0.285	-14.860	0.285	-14.785	0.288	-12.056
0.305	-14.872	0.305	-14.853	0.305	-14.825	0.305	-14.745	0.309	-10.928
0.325	-14.837	0.325	-14.818	0.325	-14.788	0.325	-14.699	0.330	-9.510
0.345	-14.800	0.345	-14.779	0.345	-14.747	0.345	-14.645	0.352	-7.882
0.365	-14.760	0.365	-14.737	0.365	-14.700	0.365	-14.579	0.374	-6.202
0.385	-14.714	0.385	-14.687	0.385	-14.645	0.386	-14.494	0.395	-4.644
0.405	-14.658	0.405	-14.627	0.405	-14.576	0.406	-14.378	0.417	-3.335
0.425	-14.588	0.425	-14.550	0.426	-14.484	0.426	-14.210	0.438	-2.320
0.446	-14.491	0.446	-14.441	0.446	-14.352	0.446	-13.958	0.458	-1.579
0.466	-14.346	0.466	-14.277	0.466	-14.151	0.466	-13.571	0.479	-1.060
0.486	-14.112	0.486	-14.015	0.486	-13.831	0.487	-12.975	0.499	-0.704
0.506	-13.714	0.506	-13.572	0.507	-13.303	0.508	-12.066	0.520	-0.462
0.527	-13.009	0.527	-12.805	0.528	-12.418	0.529	-10.719	0.540	-0.298
0.548	-11.741	0.549	-11.460	0.549	-10.935	0.551	-8.817	0.560	-0.184
0.571	-9.459	0.571	-9.117	0.572	-8.502	0.574	-6.284	0.580	-0.103
0.595	-5.425	0.595	-5.138	0.595	-4.645	0.597	-3.101	0.600	-0.043
0.621	1.447	0.621	1.348	0.621	1.185	0.621	0.727	0.620	0.007
0.653	12.584	0.651	11.386	0.650	9.572	0.645	5.270	0.640	0.051
0.690	29.730	0.686	26.180	0.681	21.172	0.671	10.760	0.660	0.095
0.735	54.875	0.727	47.104	0.717	36.836	0.698	17.652	0.680	0.143
0.790	90.453	0.776	75.967	0.758	57.860	0.727	26.655	0.700	0.197

Fig. 1(c)

$N_d=1\times10^{20}$ cm^{-3}									
$D_i=1\times10^{12}$ cm^{-2}		$D_i=2\times10^{12}$ cm^{-2}		$D_i=5\times10^{12}$ cm^{-2}		$D_i=1\times10^{13}$ cm^{-2}		$D_i=2\times10^{13}$ cm^{-2}	
Voltage (V)	J (mA/cm^2)	Voltage (V)	J (mA/cm^2)	Voltage (V)	J (mA/cm^2)	Voltage (V)	J (mA/cm^2)	Voltage (V)	J (mA/cm^2)
-0.015	-15.352	-0.015	-15.344	-0.015	-15.311	-0.015	-15.221	-0.015	-14.784
0.005	-15.332	0.005	-15.323	0.005	-15.289	0.005	-15.197	0.005	-14.742
0.025	-15.311	0.025	-15.302	0.025	-15.268	0.025	-15.174	0.025	-14.696
0.045	-15.290	0.045	-15.281	0.045	-15.246	0.045	-15.150	0.045	-14.642
0.065	-15.269	0.065	-15.259	0.065	-15.223	0.065	-15.125	0.065	-14.576
0.085	-15.247	0.085	-15.237	0.085	-15.200	0.085	-15.100	0.086	-14.492
0.105	-15.224	0.105	-15.214	0.105	-15.177	0.105	-15.074	0.106	-14.376
0.125	-15.201	0.125	-15.191	0.125	-15.153	0.125	-15.047	0.126	-14.213
0.145	-15.178	0.145	-15.167	0.145	-15.128	0.145	-15.020	0.146	-13.972
0.165	-15.154	0.165	-15.143	0.165	-15.103	0.165	-14.992	0.166	-13.613
0.185	-15.129	0.185	-15.118	0.185	-15.077	0.185	-14.962	0.187	-13.079
0.205	-15.104	0.205	-15.092	0.205	-15.050	0.205	-14.932	0.208	-12.302
0.225	-15.078	0.225	-15.066	0.225	-15.023	0.225	-14.901	0.229	-11.224
0.245	-15.051	0.245	-15.039	0.245	-14.995	0.245	-14.868	0.250	-9.831
0.265	-15.023	0.265	-15.011	0.265	-14.965	0.265	-14.834	0.272	-8.191
0.285	-14.995	0.285	-14.983	0.285	-14.935	0.285	-14.797	0.294	-6.460
0.305	-14.966	0.305	-14.953	0.305	-14.904	0.305	-14.757	0.315	-4.830
0.325	-14.935	0.325	-14.922	0.325	-14.871	0.325	-14.712	0.337	-3.449
0.345	-14.903	0.345	-14.889	0.345	-14.836	0.345	-14.660	0.358	-2.378
0.365	-14.869	0.365	-14.855	0.365	-14.798	0.365	-14.598	0.378	-1.601
0.385	-14.832	0.385	-14.816	0.385	-14.755	0.385	-14.518	0.399	-1.062
0.405	-14.789	0.405	-14.773	0.405	-14.706	0.406	-14.409	0.419	-0.699
0.425	-14.738	0.425	-14.720	0.425	-14.644	0.426	-14.252	0.440	-0.457
0.445	-14.670	0.445	-14.650	0.445	-14.561	0.446	-14.018	0.460	-0.298
0.465	-14.570	0.465	-14.547	0.466	-14.438	0.466	-13.658	0.480	-0.192
0.486	-14.409	0.486	-14.380	0.486	-14.239	0.487	-13.097	0.500	-0.123
0.506	-14.123	0.506	-14.087	0.506	-13.895	0.508	-12.231	0.520	-0.077
0.526	-13.585	0.526	-13.537	0.527	-13.269	0.529	-10.926	0.540	-0.047
0.547	-12.536	0.548	-12.471	0.548	-12.102	0.551	-9.046	0.560	-0.028
0.570	-10.455	0.570	-10.373	0.570	-9.905	0.574	-6.491	0.580	-0.014
0.594	-6.323	0.594	-6.247	0.594	-5.816	0.597	-3.223	0.600	-0.006
0.622	1.761	0.622	1.735	0.622	1.584	0.621	0.758	0.620	0.001
0.657	17.110	0.657	16.678	0.654	14.392	0.646	5.507	0.640	0.005
0.705	45.299	0.704	43.637	0.695	35.482	0.671	11.233	0.660	0.009
0.775	95.097	0.770	90.226	0.748	68.412	0.698	18.371	0.680	0.012

Fig. 2(a)

$N_d = 1\times10^{18}$ cm^{-3}				
D_i (cm^{-2})	FF (%)	Efficiency (%)	V_{oc} (V)	J_{sc} (mA/cm^2)
1×10^{10}	67.02	11.94	0.6164	15.17888
1×10^{11}	65.95	11.74	0.6164	15.16704
5×10^{11}	59.3	10.5	0.6164	15.09632
1×10^{12}	45.77	8.03	0.6167	14.94877
2×10^{12}	7.97	1.12	0.6175	11.96622

Fig. 2(b)

$N_d = 1\times10^{19}$ cm^{-3}				
D_i (cm^{-2})	FF (%)	Efficiency (%)	V_{oc} (V)	J_{sc} (mA/cm^2)
1×10^{11}	73.75	13.22	0.6165	15.27233
5×10^{11}	73.06	13.09	0.6165	15.26004
1×10^{12}	71.87	12.86	0.6165	15.24198
2×10^{12}	67.71	12.07	0.6165	15.19383
5×10^{12}	37.84	6.61	0.6171	14.87958

Fig. 2(c)

$N_d = 1\times10^{20}$ cm^{-3}				
D_i (cm^{-2})	FF (%)	Efficiency (%)	V_{oc} (V)	J_{sc} (mA/cm^2)
1×10^{12}	75.87	13.66	0.6166	15.33651
2×10^{12}	75.69	13.62	0.6166	15.32766
5×10^{12}	74.66	13.4	0.6165	15.29419
1×10^{13}	68.19	12.17	0.6165	15.20299
2×10^{13}	28.28	4.9	0.6173	14.75345

Fig. 3(b)

$N_d=1\times10^{18}$ cm^{-3}		$N_d=1\times10^{19}$ cm^{-3}		$N_d=1\times10^{20}$ cm^{-3}	
D_i (cm^{-2})	CIGS band bending (eV)	D_i (cm^{-2})	CIGS band bending (eV)	D_i (cm^{-2})	CIGS band bending (eV)
1×10^{10}	0.50077	1×10^{11}	0.50077	1×10^{12}	0.50077
1×10^{11}	0.49285	5×10^{11}	0.49285	2×10^{12}	0.49285
5×10^{11}	0.44775	1×10^{12}	0.44775	5×10^{12}	0.44775
1×10^{12}	0.36499	2×10^{12}	0.36499	1×10^{13}	0.36499
2×10^{12}	0.13066	5×10^{12}	0.13066	2×10^{13}	0.13066

Fig. 3(c)

$N_d=1\times10^{18}$ cm^{-3}		$N_d=1\times10^{19}$ cm^{-3}		$N_d=1\times10^{20}$ cm^{-3}	
D_i (cm^{-2})	Efficiency (%)	D_i (cm^{-2})	Efficiency (%)	D_i (cm^{-2})	Efficiency (%)
1×10^{10}	11.94	1×10^{11}	13.22	1×10^{12}	13.66
1×10^{11}	11.74	5×10^{11}	13.09	2×10^{12}	13.62
5×10^{11}	10.5	1×10^{12}	12.86	5×10^{12}	13.4
1×10^{12}	8.03	2×10^{12}	12.07	1×10^{13}	12.17
2×10^{12}	1.12	5×10^{12}	6.61	2×10^{13}	4.9
